# Parental experiences and opinions regarding the management of acute otitis media in Finland—a comparative questionnaire between 2006 and 2019

**DOI:** 10.1093/fampra/cmad069

**Published:** 2023-06-30

**Authors:** Sylvia Jokinen, Aino Ruohola, Paula A Tähtinen

**Affiliations:** Department of Paediatrics and Adolescent Medicine, Turku University Hospital and University of Turku, Turku, Finland; Department of Paediatrics and Adolescent Medicine, Turku University Hospital and University of Turku, Turku, Finland; Department of Paediatrics and Adolescent Medicine, Turku University Hospital and University of Turku, Turku, Finland

**Keywords:** antibiotics, analgesics, opinions, otitis media, parents

## Abstract

**Background:**

Treatment guidelines for acute otitis media (AOM) have changed over the past 20 years. Watchful waiting is often recommended as an option for antibiotic treatment and the use of proper pain medication is emphasised.

**Objective:**

To study parental experiences and opinions regarding the management of AOM and compare our findings with our previous questionnaire submitted in 2006.

**Methods:**

We sent an online survey link through day-care centres and Facebook parental groups in Turku area. Children <4 years of age attending day care were included in the analysis. We asked about the child’s history of AOM, parental opinions about AOM treatment, and antibiotic resistance. Results of 2019 were compared with those of 2006.

**Results:**

Altogether 84% (320/381) and 83% (568/681) of children had had at least 1 episode of AOM in 2019 and 2006, respectively. In 2019, more children had been treated without antibiotics (30% vs. 13%, *P* < 0.001) and fewer parents thought that antibiotics are necessary for the treatment of AOM (70% vs. 85%, *P* < 0.001) compared with 2006. The use and knowledge of painkillers had increased over the past 13 years. Painkillers had been given at least once to 93% (296/320) of children in 2019 and 80% (441/552) of children in 2006 (*P* < 0.001).

**Conclusions:**

Today, more parents accept watchful waiting as a treatment option for AOM and give painkillers to their children, which indicates that the education about optimal management of AOM has reached parents.

**Key messages**

- The number of children treated without antibiotics has increased in Finland.
- Nowadays more parents accept watchful waiting as a treatment option for acute otitis media (AOM).
- Painkillers have been given to children more often than before during AOM.

## Introduction

Acute otitis media (AOM) is a common infection in young children and one of the most common reasons for doctoral visits.^1^ By the age of 3 years, 60% of children have had at least 1 episode of AOM and 24% at least 3 episodes of AOM.^2^ Guidelines for the treatment of AOM vary internationally. Guidelines mainly differ in terms of which patients need antibiotic treatment and when it is the right time to initiate antibiotics.^3–5^ The Finnish AOM treatment guideline primarily recommends antibiotics for all children with certain diagnosis of AOM. If a watchful waiting strategy is chosen, the guideline recommends a revisit after 2–3 days if there is no defined improvement in the child’s condition.^6^

Over the past 10 years, treatment guidelines have become more favourable to the watchful waiting strategy. Previous studies have shown that antibiotic treatment accelerates the recovery of AOM.^7^ However, some patients recover well without antibiotics, and adverse events and nasopharyngeal carriage of resistant strains of bacteria are more common in patients treated with antibiotics.^7–9^ Discussion of adverse events of antibiotics and bacterial resistance has increased.^10^ This has presumably also influenced parental opinions about antibiotic treatment.

Our aim was to study parental experiences and opinions regarding the management of AOM in Finland. We also compared our findings with our previous questionnaire submitted in 2006 to see if experiences and opinions about the use of painkillers and antibiotics in AOM have changed in 13 years.^11^

## Methods

The study was carried out in the Department of Paediatrics and Adolescent Medicine, Turku University Hospital, and the University of Turku. We collected the data through an online survey using the Webropol tool. Parents were able to complete the survey from March to July 2019. The questionnaire was sent as an electronical link through day-care centres, Facebook parental groups, and the study website in Turku region (Turku, Kaarina, Lieto, or Raisio). We also delivered advertisements to local well-baby clinics. Parents were asked to fill in the information for their oldest child, aged under 4 years. In the questionnaire, we asked about the child’s history of AOM and parental opinions regarding the management of AOM and antibiotic resistance. The questions are presented in Table 2. As a comparison, we had data from our previous questionnaire submitted in 2006.^11^

Children less than 4 years of age who attended day care and lived in Turku region were included in the study. Thus, the admission criteria were identical with the criteria in 2006. The Webropol tool was set to allow the opening of the questionnaire only once from the same browser to avoid multiple answers from the same parent.

When the first questionnaire was sent in 2006, the Finnish treatment guideline from year 2004 recommended to treat AOM with antibiotics (amoxicillin 40 mg/kg/day for 5–7 days) in virtually all children. There was also an option for watchful waiting, in which case the child had to be re-examined after 1–2 days.^12^ The current AOM treatment guideline from 2017 still recommends to treat AOM with antibiotics (amoxicillin 40 mg/kg/day or amoxicillin-clavulanate 40/5.7 mg/kg/day for 5–7 days) in all children regardless of their age if the diagnosis is certain. If watchful waiting is chosen, a child needs to be re-examined only if he/she is not clearly recovering.^6^ The recommended regimens for pain medication were per oral paracetamol, ibuprofen, and naproxen in 2004 and 2017.

Statistical analyses were performed using SPSS Statistics version 26 (IBM Corporation, Armonk, NY) software. Questionnaire data were summarised using descriptive statistics, that is frequencies per question. Percentage differences with 95% confidence intervals (CIs) were calculated to compare the 2006 and 2019 results.

## Results

**Fig. 1.**
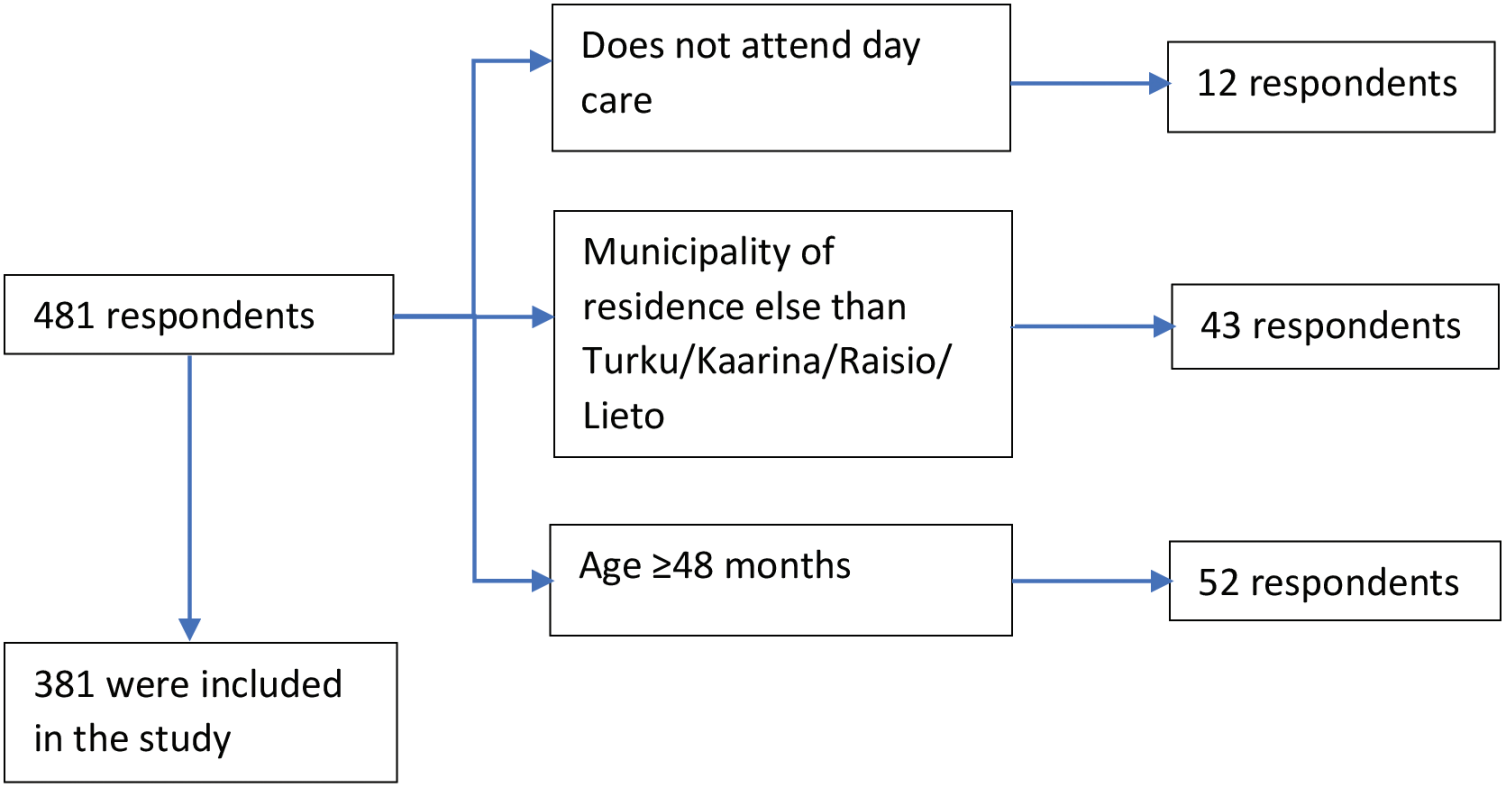
Answers included in the analysis and the exclusion criteria. Some of the children filled more than 1 exclusion criteria.

In 2019, we received a total of 481 answers, of which 381 met the inclusion criteria (day care, age, and hometown) and were thus included into the analysis (Fig. 1). In 2006, we received 1,429 answers, of which 686 concerned children under 4 years of age and were included in the analysis.

In 2019, 84 % (320/381) of children had had at least 1 episode of AOM, so these families answered to questions regarding the treatment of past AOM cases. The baseline characteristics of the 2006 and 2019 study populations were similar (Table 1), with the exception of a significantly higher maternal education level in 2019 (*P* < 0.001).

**Table 1.** Background data.

	2019	2006	*P* value
Age, median in months (range)	32 (9–47)	33 (10–47)	
Gender			
Boy	51% (194/381)	53% (300/568)	0.41
Girl	49% (186/381)	47% (268/568)	
Other	0% (1/381)	0% (0/568)	
Number of children in the household			
1	39% (150/381)	47% (323/685)	0.045
2–3	57% (217/381)	50% (343/685)	
≥4	4% (14/381)	3% (19/685)	
Number of ear infections in total			
0	16% (61/381)	17% (118/685)	0.022
1–3	45% (173/381)	37% (253/685)	
≥4	39% (147/381)	46% (314/685)	
Number of ear infections in last year			
0	27% (87/320)	22% (121/562)	0.023
1–3	57% (183/320)	56% (315/562)	
≥4	16% (50/320)	22% (126/562)	
Education of the mothers			
Lower or middle education^1^	34% (129/381)	65% (436/672)	<0.001
Higher education^2^	66% (252/381)	35% (236/672)	

^1^Comprehensive school, upper secondary school, vocational school.

^2^University of applied sciences or university.

Antibiotics were used at least once for the treatment of AOM in 96% (308/320) of children in 2019 and in 99% (558/563) of children in 2006 (rate difference [RD] −3%, 95% CI: −5% to −1). AOM was treated by watchful waiting more frequently in 2019 than in 2006, 30% (95/320) versus 13% (70/551), respectively (RD 17%, 95% CI: 12% to 22%). In 2019, 70% (223/320) and in 2006, 85% (383/450) of parents thought that their child’s ear infection needed antibiotics to heal (RD 15%, 95% CI: −21% to −10%).

The use of painkillers had increased over the past 13 years (Table 2). Painkillers were recommended at least once by a nurse or doctor to 91% (290/320) of parents in 2019 and 77% (415/539) of parents in 2006 (RD 14%, 95% CI: 8% to 19%). Painkillers had been given at least once to 93% (296/320) of children in 2019 and 80% (441/552) of children in 2006 (RD 13%, 95% CI: 8% to 18%).

**Table 2.** Antibiotics and analgesic use during AOM

	“Yes” 2019	“Yes” 2006	Rate difference (95% Confidence interval)	*P* value
Has the doctor ever prescribed antibiotics to treat ear infection of your child?	96% (308/320)	99% (558/563)	−3% (−5% to −1)	0.003
Has the doctor ever treated the ear infection of your child without antibiotics but with watchful waiting?	30% (95/320)	13% (70/551)	17% (12% to 22%)	<0.001
Do you think that antibiotics are necessary in the treatment of the ear infection of your child?	70% (223/320)	85% (383/450)	−15% (−21% to −10%)	<0.001
Has a general practitioner ever taken care of the ear infection of your child?	35% (113/320)	60% (338/561)	−25% (−32% to −18%)	<0.001
Has a private practitioner ever taken care of the ear infection of your child?	77% (245/320)	68% (383/561)	8% (2% to 15%)	0.009
Has a doctor or nurse ever recommended using painkillers in connection to the ear infection?	91% (290/320)	77% (415/539)	14% (8% to 19%)	<0.001
Have you ever given painkillers to your child in connection to the ear infection?	93% (296/320)	80% (441/552)	13% (8% to 18%)	<0.001
When do you think it is important to give your child painkillers? (You can choose several answers)				
When I, as a parent, suspect that my child has an ear infection.	41% (157/381)	21% (143/681)	20% (15% to 26%)	<0.001
After the doctor has diagnosed an ear infection	40% (152/381)	24% (162/681)	16% (10% to 22%)	<0.001
When ear infection is treated with antibiotics	26% (98/381)	10% (69/681)	16% (11% to 20%)	<0.001
When ear infection is treated without antibiotics	31% (118/381)	21% (144/681)	10% (4% to 15%)	<0.001
Only when my child seems to be in pain	69% (262/381)	84% (571/681)	−15% (−20% to −10%)	<0.001

Altogether 39% (147/381) and 37% (252/675) of parents had discussed bacterial resistance to antibiotics with their doctor in 2019 and in 2006, respectively (RD 1%, 95% CI −5% to 7%). In 2019, 43% (164/381) and in 2006, 48% (314/661) of parents had heard that unnecessary use of antibiotics increases bacterial resistance (RD −5%, 95% CI −11 to 2%). In 2019, 86% (327/381) and in 2006, 87% (572/659) of parents were concerned about bacterial resistance to antibiotics (RD −1%, 95% CI: −5% to 3%). In addition, 35% (132/381) of parents in 2019 and 34% (211/613) in 2006 believed that if a course of antibiotics is taken to the end, the bacteria would not become resistant to antibiotics (RD 0%, 95% CI −6% to 6%). In 2019, 11% (36/320) of parents reported having had problems with bacterial resistance when treating a child’s ear infection, compared to 20% (131/643) in 2006 (RD −9%, 95% CI: −14% to −4%).

In the subgroup analyses, the results were similar in children less than 24 months of age and children older than 24 months of age (data not shown).

## Discussion

Our results show that attitudes toward management of AOM without antibiotics have become more acceptable in Finland. This may have been influenced by research data on the safety and efficacy of watchful waiting strategy.^7,8,13^ The first Finnish AOM treatment guideline from 1999^14^ and its update in 2004^12^ already provided the possibility of treating a child with watchful waiting if the child was re-examined after 2–3 days. Despite this, only 13% of parents reported that their child had been treated without antibiotic in 2006. The current AOM treatment guideline from 2017 is more flexible, suggesting that a child needs to be re-examined only if he/she is not clearly recovering.^6^ The Finnish AOM treatment guideline is still more in favour of antibiotics than those of many other countries.^3,15^ The biggest change may have been in attitudes towards antibiotics, both among doctors and parents. Markedly more children were treated with watchful waiting in 2019 compared to 2006. In addition, fewer parents thought that antibiotics would be necessary to treat AOM in 2019 than in 2006, although a majority still preferred antibiotic treatment. In a German study,^16^ an even higher proportion (92.5%) of parents felt that AOM needs antibiotics to heal, and in Australia,^17^ 92% of parents thought that antibiotics are beneficial in treating AOM. On the other hand, approximately 40% of German parents responded that an antibiotic is not needed until symptoms have lasted 2 days or become more severe, which is in line with treatment guidelines in various countries.^16^

The watchful waiting strategy can be chosen by a mutual decision between a doctor and a parent. Almost all parents would like to be involved in the decision-making.^18^ A recent US study of more than 2 million AOM episodes found that paediatricians are less likely than otolaryngologists to choose the watchful waiting strategy.^19^ Clinicians should give parents the option of choosing treatment without antibiotics. Therefore, it would be important for parents to have basic knowledge of ear infection, the treatment options, and changing treatment recommendations.

Antibiotic use increases bacterial resistance. In 2006, resistance had caused more problems in Finland than in Netherlands, and antibiotic use was more common in Finland than in the Netherlands^.11^ In 2019, fewer parents reported having had problems with antibiotic resistance when treating their child’s AOM compared to 2006, which may reflect a decrease in the number of penicillin-resistant strains in Finland and increased parental knowledge about antibiotic resistance. The 10-valent pneumococcal conjugate vaccine (PCV) has been in the Finnish national vaccine programme since 2010. The vaccination coverage is good, and over 90% of children born after 2012 have at least 2 doses of PCV.^20^ Since the introduction of PCV in the national vaccine program, the numbers of both, penicillin-resistant pneumococci and invasive pneumococcal infections, have decreased significantly in Finland.^21^ Based on our results, PCVs and decreased resistance rates may have had a significant impact on the treatment of AOM as well.

We also found that the use of painkillers in the management of AOM had increased. A significantly higher proportion of parents responded in 2019 that they had given their child painkillers at least once during AOM than in 2006. Several studies have shown that it is very difficult for parents to estimate their child’s ear pain, especially in young preverbal children.^22,23^ It is therefore important for parents to give painkillers to children as soon as they suspect AOM.

In 2006, we found some alarming results about parental awareness of painkillers, as only 21% of parents reported giving painkillers when they suspected their child to have AOM.^11^ Now again, a higher proportion of parents responded that giving painkillers to their child was important in the event of AOM. Yet, 69% of parents answered that it is necessary to give painkillers only if the child seemed to be in pain. Health professionals should therefore continue to focus on providing good instructions on the use of painkillers during AOM and encourage parents to treat pain as soon as they suspect their child has AOM.

A significantly higher proportion of parents had received guidance on pain medication in the treatment of AOM in 2019 than in 2006. This indicates that the guidance provided by healthcare professionals has increased and improved so that parents now remember the guidance and use it for the benefit of their child. According to a Dutch study, parents rely on physicians’ preferences and are more confident in treating their child independently during the next AOM episode if they have received good instructions on pain medication from their physician.^24^ It is therefore important for physicians to guide parents on painkiller dosing and regular dosing intervals.

The major strength of this study is the comparison of parents’ experiences of AOM management with identical set of questions in the same setting 13 years apart. The study was carried out in spring and summer so that parents could remember well their experiences with their child’s AOM. In addition, the 2006 and 2019 study populations were very similar in terms of age and gender distribution of children.

This study has some limitations. First, the survey was based on parents’ recollections of children’s AOM history, so recall bias may have occurred. Second, maternal education levels were significantly higher in 2019 than in 2006. The questionnaire was distributed and collected in paper form through day-care centres in 2006, which presumably put pressure on parents to respond. In 2019, day-care centres shared information about the study to parents via e-mail and the data were collected as an internet survey. Presumably, parents with higher education were more willing to respond to the voluntary survey. It is possible that knowledge of the effects of antibiotics and pain medication has reached the educated better than the less educated, which may have affected the results. However, it can be stated that in Finland, everybody has equal access to healthcare services and healthcare professionals share the same information to all parents.

## Conclusions

Changes in treatment guidelines seem to affect treatment practices and parental opinions. Today, more parents than before accept watchful waiting as a treatment option for AOM and give painkillers to their children, indicating that the education on optimal management of AOM has reached parents. However, more work needs to be done to increase the knowledge about antibiotic resistance. Through good co-operation with parents, we can transfer the knowledge to families and thus improve the care of children.

## Supplementary Material

cmad069_suppl_Supplementary_Checklist

## Data Availability

The data underlying this article will be shared on reasonable request to the corresponding author.
